# The Obstetrical Extractor

**Published:** 1850-08

**Authors:** 


*The Obstetrical Extractor.* A paper read before the Chicago Medical Society. By JOHN EVANS, M. D., Prof. of Obstetrics in the Rush Medical College, etc., etc.

This is the title of a memoir communicated for the North Western Medical and Surgical Journal, by Prof. Evans, one of the editors of that periodical, and also issued as a separate publication. Prof. E. submitted his new instrument at the late meeting of the American Medical Association, explaining the principles of its adaptation, and replying to such objections as were suggested by different members. The mode of its application, also, at the same time, was demonstrated with the manakin. Like most discoverers, or inventors, he is sanguine in the belief that the *Extractor* will be found to be greatly superior to the forceps in most cases in which mechanical aid is required to effect delivery, and we sincerely hope that these expectations will be realized. Leaving for others more conversant with such matters than ourselves, critical investigation of the merits of the instrument, we transfer to our columns all the article referred to, except the plates, and what relates thereto, which, of course, we are obliged to omit.—EDITOR BUFFALO MEDICAL JOURNAL.

The idea of laying hold of the head of the child, to exert extractive force upon it, must have occurred to the first intelligent accoucher in attendance upon a tedious or obstructed labour. And yet we have no account of any successful means having been devised for fulfilling this plain indication, which so frequently occurs in the practice of every Obstetrician, up to the time of the Chamberlains. And since the discovery and universal adoption of the obstetrical forceps, which, by general consent, are not proper to be used in one-fourth the cases in which this kind of aid is indicated, the whole profession has, strangely enough, seemed to be satisfied with efforts at improving them, without seeming to think a better contrivance possible or desirable. So much so has this been the case, that when two years ago, I proposed to devise an instrument that would be simpler, safer, and more general in its application, I was met on every hand by the reply, that the forceps, properly constructed, were all that was wanted. But the multiplicity of modifications through which the forceps have gone, shows that there is great deficiency in the instrument.

Since the greatest obstacle in the way of improvement, is a want of a proper understanding of the errors and deficiencies under which we labor, I will, before describing the instrument I have invented, and used as a means of extracting the head of the child in difficult labor, direct attention to some of the numerous defects of the forceps. For, notwithstanding they are the greatest triumph of obstetrical art, if not the most useful instrument ever invented for the relief of suffering humanity; still, they fall far short of fulfilling all the indications for a resort to extractive force in parturition.

The size of the forceps, with their wide, rigid, permanently curved clamps, renders their introduction difficult; in all cases painful — often dangerous — and under many circumstances, entirely impossible. The dangers of lacerating the vagina, os tincæ and perinæum, and of destroying the child, from compression of the head, where much force is required, are familiar to all, and universally admitted.

A large number of cases evidently demanding such assistance as they give, are allowed to go on hour after hour, and sometimes, even days, without relief, from a horror of applying the forceps; which can only be the result of their danger, and the pain necessarily inflicted by them.

The forceps are not applicable until the head has engaged in the superior strait, and according to British authors, not until it has descended into the cavity of the pelvis, and are not applicable until the os tincæ is dilated, under any circumstances. Hence, they are useless in many cases of hemorrhage and convulsions, demanding speedy delivery.

They interfere with the natural rotation, if applied high up in the pelvis, and thus render the delivery more difficult.

The rules for the application of the forceps are so complex and varied, that they are learned and understood with difficulty, and the cases in which their use is recommended occur so rarely, that few practitioners are prepared to use them, when the emergency requiring it comes.

Certainly, under these circumstances, and in view of the great amount of suffering endured in consequence of the want of an instrument to fulfil the indications in these and other cases, it will not be out of place to give the results of my efforts to furnish the desideratum.

*The Obstetrical Extractor* was exhibited to my class in Rush Medical College during the winter of 1848–9; but, by waiting to test its applicability, it has not been made more public until now.

The principle upon which it operates is plain and simple, being that of placing a band or fillet around the head of the child above its largest diameter, and fixing the ends near together, by steel fingers, so that it cannot be drawn off. From this band straps pass down to the vertex and out through the os externum, to be grasped by the hand, and upon which the extractive force is exerted.

The band or fillet is applied after the manner of passing the ligature around a polypus by Gooch's double canula, as will be more readily understood after describing the instrument.

The Extractor consists of two blades or fingers of steel that are exactly alike; each 11 inches long, 5 inches of which is handle and six inches finger; a sliding ring; a silk band passing from one finger to the other, 9 inches long by 1 inch wide, and 4 straps of silk braid passing from it downward between the fingers, 18 inches long, and one-third of an inch wide. Between these straps, and attached to the inferior margin of the band is a net work two inches wide, made of silk braid.

The handles are five-eighths of an inch wide, and three-sixteenths thick, uniform and straight the entire length, so that a sliding ring can be passed over both when their edges are brought together, so as firmly to fix them side by side.

The other part forms the finger which has a curvature to fit the convexity of the child's head. It is one-fourth of an inch thick at the junction with the handle, for here the greatest strain comes on the instrument, but rapidly becomes thinner toward the end, until it is as much attenuated as it can be, to allow of two strong hinge joints in it. It tapers also in width from five-eighths of an inch at the handle to three-eighths at the end, where it is terminated by a ring, the hole through which is one-half of an inch in diameter. The edges of the fingers are rounded, and the hinges made so that they present no roughness. They allow flexion enough to adapt the finger to any part of the head upon which it may rest at the time, and extension enough to make it as straight as is desirable in the introduction of the instrument. The slide holds the handle and fingers parallel and firmly together, so that their ends cannot separate.

The band or fillet is firmly attached to the end of each finger at the ring, and by a few drilled holes below it. This is the main part of the instrument, as the steel fingers are only used to pass it around the head and hold it there.

The net work is designed to keep the band from slipping over the chin of the child, and to keep the straps in their places.

The straps are firmly sewed to the margin of the band nearest the handles, and pass down so as to reach some distance below the ends of the handles. These may be tied together at their ends, to form a loop in which the hand may hold when pulling to extract the child.

The thread or fine braid is to draw the band into the rings at the ends of the fingers, so that it will be carried entirely up during the introduction of the instrument.

To prepare the Extractor for application, draw the band through the rings, and tie the threads holding it to the ends of the handles; put on the slide, extend the fingers, adjust the straps, and dip the whole in oil.

To introduce it, pass two fingers of the left hand to the presenting part of the head of the child, or to the os uteri; take the instrument as prepared, by the handles, holding the straps in place, parallel with the fingers of the instrument, and pass it within the uterus, with the palmar surface of the fingers next the head. Then as it is passed up, flexion of the fingers will take place from traction on the straps, and the pressure of the uterus, so as to keep them in contact with the head, as if grasping it. With the fingers of the left hand, examine and adjust the end of the instrument, so as to give it a right direction, then gently press it up until the curve fits upon the head. When the end with its bunch of silk passes over the greatest diameter of the child's head, the resistance gives way, and it becomes free.

In this process there is no danger of injuring either mother or child, if done with moderate prudence, as the end of the instrument is protected by the bunch of silk band drawn into the rings. Next the threads that hold the band thus, are loosened, and the slide taken off. One handle is now grasped and gently carried around the head from its fellow to the opposite side. As the finger goes round, it pays out the band at the very place desired. Care must be had that none of the straps are pulled at this stage of the process, lest they draw the band too far down. The other handle is next grasped, and carried in the opposite direction until it meets its fellow. During this part of the process, two points should be attended to, to facilitate the application. First, to relieve compression between the head and any part of the pelvic walls, the former may be pressed upward. And when the instrument is passing over the occiput, if passed far up, the end of it may strike against the child's neck, and make an obstruction. If this occurs, slightly withdrawing the handle will obviate the difficulty.

When brought together, the slide is to be placed upon the handles again, and the straps adjusted with the fingers, so as to be about equidistant from each other, gathered together and passed through the staple upon the sliding ring, and drawn down tightly. Thus applied, the slide on the handles holds the fingers of the instrument parallel to each other, and fixes firmly the ends near together so that the band cannot give or slip off the head.

The instrument thus applied, is ready for the application of extractive force, which may be made as occasion requires, either directly, obliquely downward, upward, or to either side, by pulling more forcibly the opposite straps. If the head needs its position altered, it gives us the best possible power for accomplishing it.

Although I have had but limited experience in the use of the *Extractor*, so far it has proved itself to be all that I could have expected. My greatest fear, before using it, was that it would be difficult of application, and especially in passing the fingers from one side of the head to the other; but in this, even when the head has been pressed down tightly in the pelvis, I have met with comparatively little difficulty, as the whole process of application required but a minute, and scarcely caused an extra pang of suffering to the patient. But as the test of experience can scarcely be claimed for the Extractor, we will look at it philosophically, and see what indications it will probably fulfil.

As it is capable of being extended beyond a right line, it can be passed round the bulge of the head at any point in the maternal passages, and from its small size, flexibility, and smoothness, with great facility.

These traits will also enable the operator to apply it even before the os uteri is dilated larger than the size of a dollar. The bunch of silk braid at the extreme end of the instrument, made by the band being drawn into the rings, makes a perfect protection from its being pushed against the uterus or child in a way to inflict injury.

The parts applied to the head, being a kind of silken net work, are in no danger of injuring the child.

The application of force involves but little compression of the head, which is left free to be moulded to the shape of the passage.

The straps being so soft and yielding, and their adaptation to the head being so close, there is no danger of contusion or laceration resulting from them. Even when the os uteri is but slightly dillated the straps would be in no danger of injuring it.

The head is so completely grasped by the instrument, that we can apply our force so as to make but little pressure on the mother's parts, in advance of the head.

There is no precise place on the head where the instrument must be applied, as there is no danger of its injuring any part. The small size of the instrument (its weight entire is 8 ounces) renders it convenient; its simplicity understandable and safe, and its cheapness commends it especially to those who have not been able to procure the forceps, on account of their price. The extractor will probably not cost over 3 or 4 dollars when its manufacture is systemized.

The cases to which it is applicable are:

1.—All of those in which the forceps are now reccommended, excepting when the head may be so firmly locked between opposite points of the pelvis that it cannot be moved by compressing it upward.

2.—All labors protracted in the second stage, where it is possible to deliver with safety to the child. Being applied with little pain or danger there can be no excuse for allowing a patient to suffer the agonizing throes of labor, hour after hour, without progress, as is done in almost all such cases now, should the physician have the Extractor at hand.

3.—In labors obstructed at the superior strait of the pelvis, it will be especially applicable, for the higher up the head is at the time, the easier will be the application of the instrument. This will be manifest if we consider how readily it can be passed up by the side of the head when the fingers are extended, and that the only obstacle likely to interfere with its application is in passing the fingers from one side to the other, of the head. The higher the head is up, the more loosely it floats, and of course, the less resistance of this kind will be offered.

4.—Cases requiring speedy delivery at any time, either before labor has commenced, (for the os uteri toward the full time is always dilatable enough to allow of its application,) or during its progress, as is sometimes desirable in convulsions, hemorrhage, and the induction of premature labor, to prevent craniotomy.

By applying the Extractor, immediately after the separation of the placenta, in placenta previa, as recommended by Prof. Simpson, probably the head may be kept so compressed against the os uteri as to prevent fatal hemorrhage, and possibly, sometimes deliver so speedily as to save the child.

In cases of prolapsus of the funis umbilicalis, the fold may be completely returned by placing it between the ends of the fingers of the Extractor, when it will rest upon the bunch of silk band as prepared for introduction, by which it can be carried entirely above the head and held there until the head is delivered.

The difficulty of describing an instrument that is entirely unique, may prevent me from making myself understood without exhibiting it, but I think no one will fail to see its plausibility, upon an examination of the Extractor itself.

That it will fill most of the indications pointed out, I have no doubt, but extensive experience must be the test of the range of its usefulness.

*Chicago, April* 15, 1850.

